# (*E*)-4-Bromo-2-[(2-hydroxy­phen­yl)iminiometh­yl]phenolate

**DOI:** 10.1107/S1600536810015230

**Published:** 2010-05-08

**Authors:** Naser Eltaher Eltayeb, Siang Guan Teoh, Hoong-Kun Fun, Suchada Chantrapromma

**Affiliations:** a School of Chemical Sciences, Universiti Sains Malaysia, 11800 USM, Penang, Malaysia; b X-ray Crystallography Unit, School of Physics, Universiti Sains Malaysia, 11800 USM, Penang, Malaysia; c Crystal Materials Research Unit, Department of Chemistry, Faculty of Science, Prince of Songkla University, Hat-Yai, Songkhla 90112, Thailand

## Abstract

The title compound, C_13_H_10_BrNO_2_, crystallizes in a zwitterionic form. The zwitterion exists in a *trans* configuration about the C=N bond and is almost planar, the dihedral angle between the two benzene rings being 2.29 (9)°. An intra­molecular N—H⋯O hydrogen bond formed between the iminium NH^+^ and the phenolate O^−^ atoms generates an *S*(6) ring motif. In the crystal, the zwitterions are linked through O—H⋯O hydrogen bonds into chains along [101] and these chains are further connected through C—H⋯Br inter­actions into a two-dimensional network perpendicular to (101). C⋯C [3.572 (3)–3.592 (3) Å] and C⋯Br [3.5633 (19)–3.7339 (18) Å] short contacts are observed. The crystal studied was a twin with twin law 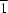
00, 0
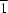
0, 001 with a domain ratio of 0.09919 (2):0.90081 (2).

## Related literature

For bond-length data, see: Allen *et al.* (1987 ▶). For hydrogen-bond motifs, see: Bernstein *et al.* (1995 ▶). For background to Schiff bases and their applications, see: Dao *et al.* (2000 ▶); Kagkelari *et al.* (2009 ▶); Karthikeyan *et al.* (2006 ▶); Sriram *et al.* (2006 ▶); Wei & Atwood (1998 ▶). For related structures, see: Eltayeb *et al.* (2009 ▶; 2010 ▶); Tan & Liu (2009 ▶). For the stability of the temperature controller used in the data collection, see Cosier & Glazer, (1986 ▶).
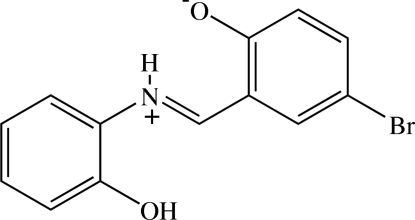


## Experimental

### 

#### Crystal data

C_13_H_10_BrNO_2_
*M* *_r_* = 291.12
Monoclinic, 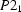

*a* = 4.6387 (3) Å
*b* = 18.9379 (13) Å
*c* = 6.2270 (4) Å
β = 90.144 (3)°
*V* = 547.02 (6) Å^3^
*Z* = 2
Mo *K*α radiation
μ = 3.74 mm^−1^
*T* = 100 K
0.43 × 0.14 × 0.14 mm

#### Data collection

Bruker APEXII DUO CCD area-detector diffractometer
Absorption correction: multi-scan (*SADABS*; Bruker, 2009 ▶) *T*_min_ = 0.295, *T*_max_ = 0.628
8575 measured reflections
3120 independent reflections
3034 reflections with *I* > 2σ(*I*)
*R*_int_ = 0.026

#### Refinement

*R*[*F* ^2^ > 2σ(*F* ^2^)] = 0.018
*wR*(*F* ^2^) = 0.041
*S* = 1.02
3120 reflections
191 parameters
1 restraint
H atoms treated by a mixture of independent and constrained refinement
Δρ_max_ = 0.59 e Å^−3^
Δρ_min_ = −0.29 e Å^−3^
Absolute structure: Flack (1983 ▶), 1480 Friedel pairs
Flack parameter: 0.027 (7)

### 

Data collection: *APEX2* (Bruker, 2009 ▶); cell refinement: *SAINT* (Bruker, 2009 ▶); data reduction: *SAINT*; program(s) used to solve structure: *SHELXTL* (Sheldrick, 2008 ▶); program(s) used to refine structure: *SHELXTL*; molecular graphics: *SHELXTL*; software used to prepare material for publication: *SHELXTL* and *PLATON* (Spek, 2009 ▶).

## Supplementary Material

Crystal structure: contains datablocks global, I. DOI: 10.1107/S1600536810015230/rz2436sup1.cif

Structure factors: contains datablocks I. DOI: 10.1107/S1600536810015230/rz2436Isup2.hkl

Additional supplementary materials:  crystallographic information; 3D view; checkCIF report

## Figures and Tables

**Table 1:** Hydrogen-bond geometry (Å, °)

*D*—H⋯*A*	*D*—H	H⋯*A*	*D*⋯*A*	*D*—H⋯*A*
O2—H1*O*2⋯O1^i^	0.82	1.76	2.5641 (19)	169
N1—H1*N*1⋯O1	0.89 (3)	1.84 (3)	2.6129 (18)	143 (3)
C7—H7*A*⋯O2	0.95 (2)	2.12 (2)	2.794 (2)	127.1 (18)
C11—H11*A*⋯Br1^ii^	0.96 (3)	2.89 (3)	3.6982 (19)	143.1 (19)

Symmetry codes: (i) 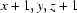; (ii) 
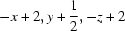
.

**Fig. 1.**
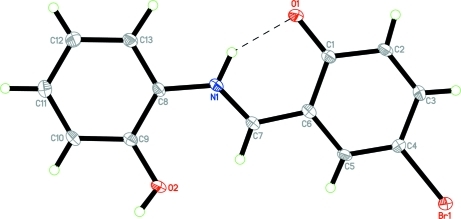
The molecular structure of the title compound, with 50% probability displacement ellipsoids and the atom-numbering scheme. Hydrogen bond is shown as dashed lines.

**Fig. 2.**
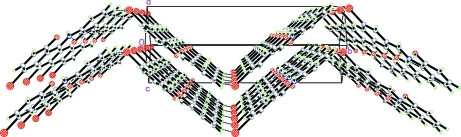
The crystal packing showing 2-D networks perpendicular to the (101)-plane.

## supplementary crystallographic information

### Comment

Much attention has been given to Schiff base ligands due to their applications such as in coordination chemistry (Kagkelari *et al.*, 2009), chelated boron catalyst (Wei & Atwood, 1998), pharmacological activities, anticancer (Dao *et al.*, 2000), anti-HIV (Sriram *et al.*, 2006), antibacterial and antifungal (Karthikeyan *et al.*, 2006) activities. We have reported the crystal structures of Schiff base ligands which existed in a zwitterionic form *i.e* 2-((*E*)-{2-[(*E*)-2,3-dihydroxybenzylideneamino]-5-methylphenyl}- iminiomethyl)-6-hydroxyphenolate (Eltayeb *et al.*, 2009) and (*E*)-4-allyl-2-{[(2-hydroxyphenyl)iminio]methyl}-6-methoxyphenolate (Eltayeb *et al.*, 2010). Herein we report the crystal structure of the title zwitterionic Schiff base ligand (I).

The molecule of (I) (Fig. 1), C_13_H_9_BrNO_2_, crystallizes in a zwitterionic form with cationic iminium and anionic enolate, and exists in a *trans* configuration about the C═N bond [1.310 (2) Å]; the torsion angle C8–N1–C7–C6 is 179.25 (17)°. The molecule is almost planar with the dihedral angle between the two benzene rings of 2.31 (9)°. The hydroxy group is co-planar with the attached C8–C13 benzene ring with the r.m.s. of 0.0102 (2) Å for the seven non H atoms. Intramolecular N—H···O hydrogen bond between the NH^+^ and the phenolate O^-^ generates an S(6) ring motif (Fig. 1; Table 1) which help to stabilize the planarity of the molecule (Bernstein *et al.*, 1995). The bond distances are in normal ranges (Allen *et al.*, 1987) and comparable with those found in related structures (Eltayeb *et al.*, 2009, 2010; Tan & Liu, 2009).

In the crystal packing (Fig. 2), the zwitterions are linked through O2–H1O2···O1 hydrogen bonds into chains along the [101] and these chains are further connected through C11—H11A···Br1 interactions into a 2-D network perpendicular to the (101)-plane. The crystal is stabilized by O—H···O and weak C—H···Br interactions (Table 1). C···C [3.572 (3)-3.592 (3) Å] and C···Br [3.5633 (19)-3.7339 (18) Å] short contacts are observed.

### Experimental

The title compound was synthesized by adding 5-bromo-2-hydroxybenzaldehyde (0.402 g, 2 mmol) to a solution of 2-aminophenol (0.218 g, 2 mmol) in ethanol (30 ml). The mixture was refluxed with stirring for half an hour. The resultant yellow solution was filtered and the filtrate was evaporated to give a yellow solid product. Yellow needle-shaped single crystals of the title compound suitable for *x*-ray structure determination were obtained from ethanol by slow evaporation at room temperature after nine days.

### Refinement

Hydroxyl H atom was placed in calculated positions with d(O—H) = 0.82 Å and the *U*_iso_ values was constrained to be 1.5*U*_eq_ of the carrier atom. The remaining H atoms were located from the difference map and isotropically refined. The highest residual electron density peak is located at 0.80 Å from Br1 and the deepest hole is located at 0.99 Å from Br1. The crystal studied was a twin with twin law 1 0 0, 0 1 0, 0 0 1, leading to a distribution (refined BASF parameter) of 0.09919/0.90081 (2).

### Crystal data

**Table:**

C_13_H_10_BrNO_2_	*F*(000) = 292
*M**_r_* = 291.12	*D*_x_ = 1.773 Mg m^−^^3^
Monoclinic, *P*2_1_	Mo *K*α radiation, λ = 0.71073 Å
Hall symbol: P 2yb	Cell parameters from 3120 reflections
*a* = 4.6387 (3) Å	θ = 1.1–30.0°
*b* = 18.9379 (13) Å	µ = 3.74 mm^−^^1^
*c* = 6.2270 (4) Å	*T* = 100 K
β = 90.144 (3)°	Needle, yellow
*V* = 547.02 (6) Å^3^	0.43 × 0.14 × 0.14 mm
*Z* = 2	

### Data collection

**Table:**

Bruker APEXII DUO CCD area-detector diffractometer	3120 independent reflections
Radiation source: sealed tube	3034 reflections with *I* > 2σ(*I*)
graphite	*R*_int_ = 0.026
φ and ω scans	θ_max_ = 30.0°, θ_min_ = 1.1°
Absorption correction: multi-scan (*SADABS*; Bruker, 2009)	*h* = −6→6
*T*_min_ = 0.295, *T*_max_ = 0.628	*k* = −26→26
8575 measured reflections	*l* = −8→8

### Refinement

**Table:**

Refinement on *F*^2^	Secondary atom site location: difference Fourier map
Least-squares matrix: full	Hydrogen site location: inferred from neighbouring sites
*R*[*F*^2^ > 2σ(*F*^2^)] = 0.018	H atoms treated by a mixture of independent and constrained refinement
*wR*(*F*^2^) = 0.041	*w* = 1/[σ^2^(*F*_o_^2^) + (0.0035*P*)^2^] where *P* = (*F*_o_^2^ + 2*F*_c_^2^)/3
*S* = 1.02	(Δ/σ)_max_ < 0.001
3120 reflections	Δρ_max_ = 0.59 e Å^−^^3^
191 parameters	Δρ_min_ = −0.29 e Å^−^^3^
1 restraint	Absolute structure: Flack (1983), 1480 Friedel pairs
Primary atom site location: structure-invariant direct methods	Flack parameter: 0.027 (7)

### Special details

**Table:**

Experimental. The crystal was placed in the cold stream of an Oxford Cryosystems Cobra open-flow nitrogen cryostat (Cosier & Glazer, 1986) operating at 100.0 (1) K.
Geometry. All esds (except the esd in the dihedral angle between two l.s. planes) are estimated using the full covariance matrix. The cell esds are taken into account individually in the estimation of esds in distances, angles and torsion angles; correlations between esds in cell parameters are only used when they are defined by crystal symmetry. An approximate (isotropic) treatment of cell esds is used for estimating esds involving l.s. planes.
Refinement. Refinement of F^2^ against ALL reflections. The weighted R-factor wR and goodness of fit S are based on F^2^, conventional R-factors R are based on F, with F set to zero for negative F^2^. The threshold expression of F^2^ > 2sigma(F^2^) is used only for calculating R-factors(gt) etc. and is not relevant to the choice of reflections for refinement. R-factors based on F^2^ are statistically about twice as large as those based on F, and R- factors based on ALL data will be even larger.

### Fractional atomic coordinates and isotropic or equivalent isotropic displacement parameters (Å^2^)

**Table:**

	*x*	*y*	*z*	*U*_iso_*/*U*_eq_	
Br1	0.00526 (4)	0.442840 (15)	0.87673 (2)	0.01554 (4)	
O1	0.5191 (3)	0.66300 (7)	0.29008 (19)	0.0152 (2)	
O2	1.1314 (3)	0.70088 (8)	1.0194 (2)	0.0166 (3)	
H1O2	1.2697	0.6925	1.0976	0.025*	
N1	0.8565 (3)	0.69973 (8)	0.6076 (2)	0.0121 (3)	
H1N1	0.802 (6)	0.6976 (16)	0.470 (5)	0.026 (7)*	
C1	0.4132 (4)	0.61426 (10)	0.4149 (3)	0.0126 (3)	
C2	0.1934 (4)	0.56695 (10)	0.3446 (3)	0.0140 (3)	
H2A	0.133 (6)	0.5756 (14)	0.207 (4)	0.020 (6)*	
C3	0.0797 (4)	0.51628 (10)	0.4781 (3)	0.0138 (3)	
H3A	−0.064 (6)	0.4852 (14)	0.440 (4)	0.021 (6)*	
C4	0.1776 (4)	0.51058 (9)	0.6919 (3)	0.0129 (3)	
C5	0.3894 (4)	0.55482 (9)	0.7685 (3)	0.0125 (3)	
H5A	0.450 (6)	0.5535 (16)	0.905 (4)	0.026 (7)*	
C6	0.5112 (4)	0.60653 (9)	0.6331 (3)	0.0122 (3)	
C7	0.7261 (4)	0.65154 (9)	0.7227 (3)	0.0123 (3)	
H7A	0.787 (5)	0.6470 (12)	0.868 (4)	0.009 (5)*	
C8	1.0707 (4)	0.74911 (9)	0.6695 (3)	0.0117 (3)	
C9	1.2064 (4)	0.74952 (9)	0.8722 (3)	0.0125 (3)	
C10	1.4149 (4)	0.80132 (10)	0.9137 (3)	0.0148 (3)	
H10A	1.494 (6)	0.8047 (15)	1.048 (5)	0.022 (6)*	
C11	1.4940 (4)	0.84982 (10)	0.7569 (3)	0.0159 (3)	
H11A	1.639 (6)	0.8847 (13)	0.786 (4)	0.018 (6)*	
C12	1.3635 (4)	0.84799 (10)	0.5548 (3)	0.0160 (3)	
H12A	1.427 (7)	0.8788 (17)	0.453 (5)	0.036 (8)*	
C13	1.1523 (4)	0.79797 (9)	0.5130 (3)	0.0139 (3)	
H13A	1.045 (5)	0.7956 (13)	0.368 (3)	0.014 (6)*	

### Atomic displacement parameters (Å^2^)

**Table:**

	*U*^11^	*U*^22^	*U*^33^	*U*^12^	*U*^13^	*U*^23^
Br1	0.01876 (7)	0.01522 (7)	0.01262 (6)	−0.00425 (9)	−0.00307 (5)	0.00324 (8)
O1	0.0161 (6)	0.0183 (6)	0.0113 (5)	−0.0031 (5)	−0.0034 (5)	0.0033 (5)
O2	0.0167 (6)	0.0224 (7)	0.0106 (6)	−0.0044 (5)	−0.0054 (5)	0.0042 (5)
N1	0.0122 (7)	0.0140 (7)	0.0102 (7)	−0.0004 (5)	−0.0033 (5)	0.0003 (5)
C1	0.0122 (7)	0.0150 (8)	0.0106 (7)	0.0001 (6)	−0.0012 (5)	−0.0002 (6)
C2	0.0156 (8)	0.0172 (8)	0.0092 (8)	−0.0007 (6)	−0.0030 (6)	−0.0007 (6)
C3	0.0136 (8)	0.0141 (8)	0.0137 (8)	−0.0022 (6)	−0.0024 (6)	−0.0018 (6)
C4	0.0142 (8)	0.0120 (7)	0.0126 (8)	−0.0007 (6)	−0.0003 (6)	0.0015 (6)
C5	0.0150 (8)	0.0137 (8)	0.0087 (8)	0.0011 (6)	−0.0030 (6)	0.0007 (6)
C6	0.0116 (7)	0.0140 (7)	0.0109 (7)	0.0014 (6)	−0.0014 (6)	−0.0021 (6)
C7	0.0123 (8)	0.0139 (8)	0.0108 (8)	0.0003 (6)	−0.0020 (6)	−0.0007 (6)
C8	0.0114 (7)	0.0114 (7)	0.0122 (7)	0.0001 (6)	−0.0033 (5)	−0.0010 (6)
C9	0.0127 (7)	0.0146 (8)	0.0103 (7)	0.0003 (6)	−0.0012 (6)	−0.0007 (6)
C10	0.0146 (8)	0.0174 (9)	0.0123 (8)	−0.0012 (6)	−0.0044 (6)	−0.0020 (6)
C11	0.0141 (8)	0.0154 (8)	0.0182 (8)	−0.0021 (7)	−0.0016 (7)	−0.0017 (6)
C12	0.0172 (9)	0.0147 (8)	0.0161 (8)	−0.0007 (7)	−0.0011 (6)	0.0022 (7)
C13	0.0128 (8)	0.0160 (8)	0.0129 (8)	0.0008 (6)	−0.0026 (6)	0.0011 (6)

### Geometric parameters (Å, °)

**Table:**

Br1—C4	1.9011 (18)	C5—C6	1.411 (2)
O1—C1	1.304 (2)	C5—H5A	0.89 (2)
O2—C9	1.346 (2)	C6—C7	1.424 (2)
O2—H1O2	0.8200	C7—H7A	0.95 (2)
N1—C7	1.310 (2)	C8—C13	1.397 (2)
N1—C8	1.417 (2)	C8—C9	1.409 (2)
N1—H1N1	0.89 (3)	C9—C10	1.401 (2)
C1—C2	1.425 (2)	C10—C11	1.391 (3)
C1—C6	1.439 (2)	C10—H10A	0.92 (3)
C2—C3	1.376 (3)	C11—C12	1.396 (3)
C2—H2A	0.91 (3)	C11—H11A	0.96 (3)
C3—C4	1.409 (2)	C12—C13	1.387 (3)
C3—H3A	0.92 (3)	C12—H12A	0.91 (3)
C4—C5	1.376 (3)	C13—H13A	1.03 (2)
			
C9—O2—H1O2	109.5	N1—C7—C6	121.74 (16)
C7—N1—C8	129.42 (16)	N1—C7—H7A	116.6 (14)
C7—N1—H1N1	111.3 (19)	C6—C7—H7A	121.6 (14)
C8—N1—H1N1	119.2 (18)	C13—C8—C9	120.02 (16)
O1—C1—C2	122.15 (15)	C13—C8—N1	115.98 (15)
O1—C1—C6	121.08 (16)	C9—C8—N1	123.97 (16)
C2—C1—C6	116.76 (16)	O2—C9—C10	122.23 (15)
C3—C2—C1	121.85 (16)	O2—C9—C8	119.39 (15)
C3—C2—H2A	125.0 (16)	C10—C9—C8	118.39 (16)
C1—C2—H2A	113.1 (17)	C11—C10—C9	121.08 (16)
C2—C3—C4	120.08 (16)	C11—C10—H10A	119.3 (18)
C2—C3—H3A	124.6 (16)	C9—C10—H10A	119.6 (17)
C4—C3—H3A	115.3 (16)	C10—C11—C12	120.15 (17)
C5—C4—C3	120.59 (16)	C10—C11—H11A	120.5 (15)
C5—C4—Br1	120.14 (13)	C12—C11—H11A	119.4 (15)
C3—C4—Br1	119.24 (13)	C13—C12—C11	119.38 (17)
C4—C5—C6	120.14 (16)	C13—C12—H12A	122 (2)
C4—C5—H5A	122 (2)	C11—C12—H12A	118 (2)
C6—C5—H5A	118 (2)	C12—C13—C8	120.94 (16)
C5—C6—C7	117.51 (15)	C12—C13—H13A	122.2 (13)
C5—C6—C1	120.57 (16)	C8—C13—H13A	116.8 (13)
C7—C6—C1	121.89 (16)		
			
O1—C1—C2—C3	−179.04 (17)	C1—C6—C7—N1	−3.8 (3)
C6—C1—C2—C3	0.1 (3)	C7—N1—C8—C13	−175.04 (17)
C1—C2—C3—C4	0.8 (3)	C7—N1—C8—C9	7.1 (3)
C2—C3—C4—C5	−0.8 (3)	C13—C8—C9—O2	−178.12 (16)
C2—C3—C4—Br1	177.31 (14)	N1—C8—C9—O2	−0.3 (3)
C3—C4—C5—C6	0.0 (3)	C13—C8—C9—C10	2.3 (3)
Br1—C4—C5—C6	−178.09 (13)	N1—C8—C9—C10	−179.92 (17)
C4—C5—C6—C7	178.93 (16)	O2—C9—C10—C11	178.26 (18)
C4—C5—C6—C1	0.8 (3)	C8—C9—C10—C11	−2.1 (3)
O1—C1—C6—C5	178.25 (17)	C9—C10—C11—C12	0.6 (3)
C2—C1—C6—C5	−0.8 (2)	C10—C11—C12—C13	0.8 (3)
O1—C1—C6—C7	0.2 (3)	C11—C12—C13—C8	−0.6 (3)
C2—C1—C6—C7	−178.85 (16)	C9—C8—C13—C12	−0.9 (3)
C8—N1—C7—C6	179.25 (17)	N1—C8—C13—C12	−178.91 (16)
C5—C6—C7—N1	178.12 (16)		

### Hydrogen-bond geometry (Å, °)

**Table:**

*D*—H···*A*	*D*—H	H···*A*	*D*···*A*	*D*—H···*A*
O2—H1O2···O1^i^	0.82	1.76	2.5641 (19)	169
N1—H1N1···O1	0.89 (3)	1.84 (3)	2.6129 (18)	143 (3)
C7—H7A···O2	0.95 (2)	2.12 (2)	2.794 (2)	127.1 (18)
C11—H11A···Br1^ii^	0.96 (3)	2.89 (3)	3.6982 (19)	143.1 (19)

Symmetry codes: (i) *x*+1, *y*, *z*+1;  (ii) −*x*+2, *y*+1/2, −*z*+2.
